# Modeling Pathologies of Diastolic and Systolic Heart Failure

**DOI:** 10.1007/s10439-015-1351-2

**Published:** 2015-06-05

**Authors:** M. Genet, L. C. Lee, B. Baillargeon, J. M. Guccione, E. Kuhl

**Affiliations:** Department of Surgery, University of California at San Francisco, San Francisco, USA; Institute for Biomedical Engineering, ETH-Zurich, Zurich, Switzerland; Department of Mechanical Engineering, Michigan State University, East Lansing, USA; Dassault Systèmes Simulia Corporation, Fremont, USA; Departments of Mechanical Engineering, Bioengineering, and Cardiothoracic Surgery, Stanford University, Stanford, USA

**Keywords:** Cardiac modeling, Hypertrophy, Growth, Hypertension, Finite element method

## Abstract

Chronic heart failure is a medical condition that involves structural and functional changes of the heart and a progressive reduction in cardiac output. Heart failure is classified into two categories: diastolic heart failure, a thickening of the ventricular wall associated with impaired filling; and systolic heart failure, a dilation of the ventricles associated with reduced pump function. In theory, the pathophysiology of heart failure is well understood. In practice, however, heart failure is highly sensitive to cardiac microstructure, geometry, and loading. This makes it virtually impossible to predict the time line of heart failure for a diseased individual. Here we show that computational modeling allows us to integrate knowledge from different scales to create an individualized model for cardiac growth and remodeling during chronic heart failure. Our model naturally connects molecular events of parallel and serial sarcomere deposition with cellular phenomena of myofibrillogenesis and sarcomerogenesis to whole organ function. Our simulations predict chronic alterations in wall thickness, chamber size, and cardiac geometry, which agree favorably with the clinical observations in patients with diastolic and systolic heart failure. In contrast to existing single- or bi-ventricular models, our new four-chamber model can also predict characteristic secondary effects including papillary muscle dislocation, annular dilation, regurgitant flow, and outflow obstruction. Our prototype study suggests that computational modeling provides a patient-specific window into the progression of heart failure with a view towards personalized treatment planning.

## Introduction

Cardiovascular disease is the leading cause of death and disability, accounting for approximately 40 % of all human mortality.^4^ Despite tremendous scientific efforts during the past 20 years, heart failure remains one of the most common, costly, disabling, and deadly medical conditions affecting more than 25 million people worldwide.^40^ Heart failure usually worsens over time; it is the major cause of hospitalization in the elderly with a 5-year mortality rate of 50%.^38^ Since disease progression is highly sensitive to various patient-specific parameters,^29^ the time line of heart failure varies significantly among affected individuals. This suggests that—for treatment outcomes to be successful long-term—treatment strategies need to be designed on an individualized, patient-specific basis.^42^

**Figure 1:**
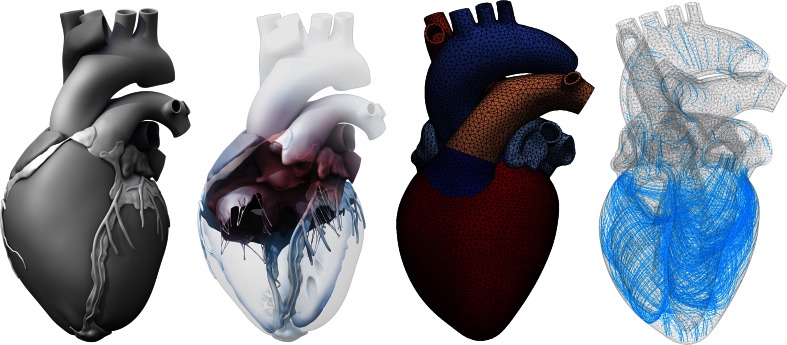
The Living Heart model. Anatomic model created from magnetic resonance images (left).^60^ Geometric model with the two atria, the two ventricles, the four valves, and the major vessels (middle, left).^60^ Finite element model with 208,561 linear tetrahedral elements and 47,323 nodes (middle, right).^5^ Muscle fiber model with 208,561 discrete fiber and sheet directions interpolated from geometric reconstruction (right).^59^

Cardiologists commonly classify heart failure into two categories, diastolic and systolic heart failure.^10^ Diastolic heart failure is associated with concentric hypertrophy, a thickening of the ventricular wall, which results in impaired filling.^21^ Systolic heart failure is associated with eccentric hypertrophy, a dilation of the ventricles, which manifests itself in reduced pump function.^16^ Both conditions ultimately result in reduced cardiac output, increased risk of cardiac arrest, and insufficient blood supply to the rest of the body.^35^

The symptoms of heart failure largely depend on which side of the heart fails.^28^ Left-sided overload, associated primarily with the systemic circulation, causes growth of the left ventricle and compromises blood flow to the body and the brain.^41^ Right-sided overload, associated with the pulmonary circulation, causes right ventricular growth and compromises blood flow to the lungs.^24^ Ultimately, both conditions can be fatal.^10^

Heart failure is a multiscale phenomenon, which gradually propagates across the scales.^9^,^48^,^58^ On the molecular level, heart failure is initiated by the parallel or serial addition or removal of sarcomeres, the smallest functional units of a heart muscle cell.^43^ On the cellular level, these alterations in cytoskeletal ultrastructure manifest themselves in a chronic thickening or lengthening of the individual muscle cells.^47^ On the whole organ level, muscle cell thickening or lengthening result in chronic ventricular wall thickening or ventricular dilation.^7^ Ultimately, these structural changes not only affect the mechanical function, but also the electrical activation of the heart.^33^

Computational modeling provides a powerful tool to reveal how local changes in cytoskeletal architecture and cellular morphology translate into global alterations of whole organ form and function.^8^,^46^ To date, numerous computational models exist to simulate the behavior of the left ventricle,^55^ a few models exist to simulate both the ventricles,^2^,^6^ but only a limited number of models exist to simulate the atria^19^ or the human heart with all four chambers.^57^ Creating whole heart models remains challenging, since the atrial wall is markedly thinner than the ventricular wall, the atria are often entangled, and their geometry can be quite complex.^19^ This not only complicates image segmentation, but also atrial discretization with an appropriate finite element mesh.

Here we create a four-chamber model of the human heart to simulate changes in cardiac form and function during diastolic and systolic heart failure. Figure 1 illustrates the underlying anatomic and geometric models created from magnetic resonance images.^60^ From this geometry, we create a finite element mesh with regionally varying muscle fiber orientations.^5^ Our model represents all four chambers as deformable, anisotropic, hyperelastic growing bodies, connected through the tricuspid and mitral valves. This allows us to predict not only the primary effects of heart failure including left and right ventricular wall thickening^21^ or dilation,^16^ but also the characteristic secondary effects including papillary muscle dislocation,^20^ mitral annular dilation,^52^ tricuspid annular dilation,^54^ regurgitant flow,^12^ and outflow obstruction.^44^ Upon appropriate calibration, our model has the potential to predict how different growth phenomena propagate across the scales with the ultimate goal to estimate the risk of heart failure and support decision-making on an individualized, patient-specific basis.

## Materials and Methods

### Continuum Model

To model cardiac growth within the framework of continuum mechanics,^3^ we multiplicatively decompose the spatial gradient ${\bf{F}}{}=\nabla _{{\bf{{X}}}{}} {\bf{\varphi }}{}$ of the deformation map ${\bf{\varphi }}{}$ into an elastic part ${\bf{F}}{}^{{{{\mathrm{{e}}}}}{}}$ and a growth part ${\bf{F}}{}^{{{{\mathrm{{g}}}}}{}}$,^53^

1 $$\begin{aligned} {\bf{F}}{} = {\bf{F}}{}^{{{{\mathrm{{e}}}}}{}} \cdot {\bf{F}}{}^{{{{\mathrm{{g}}}}}{}} \qquad \text{ with } \qquad {\bf{F}}{} = \nabla _{{\bf{{X}}}{}} {\bf{\varphi }}{}. \end{aligned}$$

Within the framework of finite growth, only ${\bf{F}}{}$ is the gradient of a continuous mapping—the elastic tensor ${\bf{F}}{}^{{{{\mathrm{{e}}}}}{}}$ and the growth tensor ${\bf{F}}{}^{{{{\mathrm{{g}}}}}{}}$ are generally associated with an incompatible configuration and do not derive as gradients from a vector field. To account for quasi-incompressibility of the elastic deformation, we further decompose the elastic tensor,

2 $$\begin{aligned} {\bf{F}}{}^{{{{\mathrm{{e}}}}}{}} = {\bf{F}}{}^{{{{\mathrm{{e}}}}}{}}_{{{{\mathrm{{vol}}}}}{}} \cdot \overline{{\bf{F}}{}}{}^{{{{\mathrm{{e}}}}}{}}\,, \end{aligned}$$

into volumetric and isochoric parts,

3 $$\begin{aligned} {\bf{F}}{}^{{{{\mathrm{{e}}}}}{}}_{{{{\mathrm{{vol}}}}}{}} = \left( J^{{{{\mathrm{{e}}}}}{}} \right) ^{1/3} {\bf{I}}{} \quad \text{ and } \quad \overline{{\bf{F}}{}}{}^{{{{\mathrm{{e}}}}}{}} = \left( J^{{{{\mathrm{{e}}}}}{}} \right) ^{-1/3} {\bf{F}}{}^{{{{\mathrm{{e}}}}}{}} \,. \end{aligned}$$

Here ${\bf{I}}{}$ denotes the second-order unit tensor, $J^{{{{\mathrm{{e}}}}}{}} = \det \left( {\bf{F}}{}^{{{{\mathrm{{e}}}}}{}} \right) =J^{{{{\mathrm{{e}}}}}{}}_{{{{\mathrm{{vol}}}}}{}}$ is the elastic Jacobian and $\overline{J^{{{{\mathrm{{e}}}}}{}}}=\det \left( \overline{{\bf{F}}{}^{{{{\mathrm{{e}}}}}{}}} \right) = 1$ is the isochoric Jacobian.^26^ As a result of this decomposition, the independent variables that characterize the elastic deformation are now the elastic Jacobian $J^{{{{\mathrm{{e}}}}}{}}$ and the isochoric deformation gradient $\overline{{\bf{F}}{}}{}^{{{{\mathrm{{e}}}}}{}}$ or, equivalently, the isochoric elastic Green–Lagrange strain tensor $\overline{{\bf{E}}{}}{}^{{{{\mathrm{{e}}}}}{}}$,

4 $$\begin{aligned} J^{{{{\mathrm{{e}}}}}{}} = \det ({\bf{F}}{}^{{{{\mathrm{{e}}}}}{}}) \;\;\; \text{ and } \;\;\; \overline{{\bf{E}}{}}{}^{{{{\mathrm{{e}}}}}{}} = \frac{1}{2} \; [\, (J^{{{{\mathrm{{e}}}}}{}})^{-2/3}\, ({\bf{F}}{}^{{{{\mathrm{{e}}}}}{}})^{{{{\mathrm{{t}}}}}{}} \!\cdot {\bf{F}}{}^{{{{\mathrm{{e}}}}}{}} - {\bf{I}}{}\,]\,, \end{aligned}$$

where the total and elastic Green–Langrage strain tensors are ${\bf{E}}{} =\frac{1}{2} \; [\, {\bf{F}}{}^{{{{\mathrm{{t}}}}}{}} \!\cdot {\bf{F}}{} - {\bf{I}}{}\,]$ and ${\bf{E}}{}^{{{{\mathrm{{e}}}}}{}} = \frac{1}{2} \; [\, ({\bf{F}}{}^{{{{\mathrm{{e}}}}}{}})^{{{{\mathrm{{t}}}}}{}} \!\cdot {\bf{F}}{}^{{{{\mathrm{{e}}}}}{}} - {\bf{I}}{}\,]$. The underlying principle of the theory of finite growth is that only elastic deformation generates stress. This implies that the strain energy function $\psi$ is a function of the elastic deformation only. To account for quasi-incompressibility, we split the strain energy function into volumetric and isochoric parts,

5 $$\begin{aligned} \psi (J^{{{{\mathrm{{e}}}}}{}}, \overline{{\bf{E}}{}}{}^{{{{\mathrm{{e}}}}}{}}) = U\left( J^{{{{\mathrm{{e}}}}}{}} \right) + \overline{\psi }\left( \overline{{\bf{E}}{}}{}^{{{{\mathrm{{e}}}}}{}} \right) \,. \end{aligned}$$

For the volumetric part, we adopt the following ansatz,

6 $$\begin{aligned} U\left( J^{{{{\mathrm{{e}}}}}{}} \right) = \frac{1}{2D_0} \left[ (J^{{{{\mathrm{{e}}}}}{}})^2 - 2\, \ln \left( J^{{{{\mathrm{{e}}}}}{}} \right) \right] \,, \end{aligned}$$

where $D_0$ controls the degree of incompressibility. For the isochoric part, we use an orthotropic Fung-type model^13^ adapted for myocardial tissue,^22^

7 $$\begin{aligned} \overline{\psi } (\overline{{\bf{E}}{}}{}^{{{{\mathrm{{e}}}}}{}}) = \frac{1}{2} \, C_0 \left[ \, \exp ( \overline{{\bf{E}}{}}{}^{{{{\mathrm{{e}}}}}{}}:{{{{\mathbf {\mathsf{{B}}}}}}}_0:\overline{{\bf{E}}{}}{}^{{{{\mathrm{{e}}}}}{}} ) - 1 \right] \,, \end{aligned}$$

keeping in mind that the growth model itself is conceptually modular and can easily be combined with alternative, more recent myocardial tissue models.^27^,^49^ In Voigt notation,^45^ the fourth-order constitutive tensor ${{\mathbf {\mathsf{{B}}}}}_0$ takes the following simple representation, $\widehat{{\bf{B}}{}_0} =\text{ diag } \left\{ B_{{{{\mathrm{{ff}}}}}{}}, B_{{{{\mathrm{{ss}}}}}{}}, B_{{{{\mathrm{{nn}}}}}{}}, 2B_{{{{\mathrm{{fs}}}}}{}}, 2B_{{{{\mathrm{{fn}}}}}{}}, 2B_{{{{\mathrm{{sn}}}}}{}} \right\}$, where the individual entries are the weights of the normal and shear strains in the fiber, sheet, and normal directions. Following the standard arguments of thermodynamics, we can then calculate the total second Piola–Kirchhoff stress,

8 $$\begin{aligned} {\bf{S}}{} = \frac{\partial \psi }{\partial {\bf{E}}{}} = \frac{\partial \psi }{\partial {\bf{E}}{}^{{{{\mathrm{{e}}}}}{}}} : \frac{\partial {\bf{E}}{}^{{{{\mathrm{{e}}}}}{}}}{\partial {\bf{E}}{}\;} =({\bf{F}}{}^{{{{\mathrm{{g}}}}}{}})^{{{{\mathrm{{-1}}}}}{}} \cdot {\bf{S}}{}^{{{{\mathrm{{e}}}}}{}} \cdot ({\bf{F}}{}^{{{{\mathrm{{g}}}}}{}})^{-{{{\mathrm{{t}}}}}{}}, \end{aligned}$$

and its elastic counterpart,

9 $$\begin{aligned} {\bf{S}}{}^{{{{\mathrm{{e}}}}}{}} = \frac{\partial \psi }{\partial {\bf{E}}{}^{{{{\mathrm{{e}}}}}{}}} \quad \text{ with } \quad {\bf{S}}{}^{{{{\mathrm{{e}}}}}{}} = {\bf{S}}{}^{{{{\mathrm{{e}}}}}{}}_{{{{\mathrm{{vol}}}}}{}} + {\bf{S}}{}^{{{{\mathrm{{e}}}}}{}}_{{{{\mathrm{{iso}}}}}{}} = \frac{\partial U}{\partial {\bf{E}}{}^{{{{\mathrm{{e}}}}}{}}} + \frac{\partial \bar{\psi }}{\partial {\bf{E}}{}^{{{{\mathrm{{e}}}}}{}}}. \end{aligned}$$

### Growth Model

To model transverse fiber growth through chronic cardiomyocyte thickening, we introduce a growth multiplier $\vartheta ^{\perp }$ that represents the parallel deposition of sarcomeres on the molecular level.^17^,^18^ The growth tensor for transverse fiber growth follows as the rank-one update of the growth-weighted unity tensor in the plane perpendicular to the fiber direction ${\bf{f}}{}_0$,

10 $$\begin{aligned} {\bf{F}}{}^{{{{\mathrm{{g}}}}}{}} = \vartheta ^{\perp } \, {\bf{I}}{} + [\,1-\vartheta ^{\perp } \,] \, {\bf{f}}{}_0 \otimes {\bf{f}}{}_0 \,. \end{aligned}$$

We invert the growth tensor using the Sherman–Morrison formula to derive an explicit expression for the elastic tensor,

11 $$\begin{aligned} {\bf{F}}{}^{{{{\mathrm{{e}}}}}{}} = \frac{1}{\vartheta ^{\perp }} \, {\bf{F}}{} + \frac{\vartheta ^{\perp }-1}{\vartheta ^{\perp }} \, {\bf{f}}{} \otimes {\bf{f}}{}_0 \,, \end{aligned}$$

where ${\bf{f}}{}={\bf{F}}{}\cdot {\bf{f}}{}_0$ denotes the fiber direction in the deformed configuration. The growth multiplier,

12 $$\begin{aligned} \vartheta ^{\perp } = \sqrt{\text{ det } ({\bf{F}}{}^{{{{\mathrm{{g}}}}}{}})} = \sqrt{J^{{{{\mathrm{{g}}}}}{}}} , \end{aligned}$$

represents the thickening of the individual muscle cells through the parallel deposition of new myofibrils. In contrast to initial growth models, which tie transverse growth ${\bf{F}}{}^{{{{\mathrm{{g}}}}}{}} = {\bf{I}}{} + [\,\vartheta ^{\perp } - 1 \,] \, {\bf{s}}{} \otimes {\bf{s}}{}_0$ to the sheet direction ${\bf{s}}{}_0$,^17^,^18^ here we interpret transverse growth as the parallel deposition of new myofibrils associated with a cross sectional area growth perpendicular to the muscle cell’s long axis ${\bf{f}}{}_0$.^58^

To model longitudinal fiber growth through chronic cardiomyocyte lengthening,^21^ we introduce a scalar-valued growth multiplier $\vartheta ^{||}$ that reflects the serial deposition of sarcomeres on the molecular level.^17^,^18^ The growth tensor for longitudinal fiber growth follows as the rank-one update of the unity tensor along the fiber direction ${\bf{f}}{}_0$,

13 $$\begin{aligned} {\bf{F}}{}^{{{{\mathrm{{g}}}}}{}} = {\bf{I}}{} + [\,\vartheta ^{||}-1\,] \, {\bf{f}}{}_0 \otimes {\bf{f}}{}_0 \,. \end{aligned}$$

Using the Sherman–Morrison formula to invert the growth tensor, we can derive the following explicit representation for the elastic tensor,

14 $$\begin{aligned} {\bf{F}}{}^{{{{\mathrm{{e}}}}}{}} = {\bf{F}}{} + \frac{1-\vartheta ^{||}}{\vartheta ^{||}} \, {\bf{f}}{} \otimes {\bf{f}}{}_0 \,. \end{aligned}$$

The growth multiplier,

15 $$\begin{aligned} \vartheta ^{||} = \text{ det } ({\bf{F}}{}^{{{{\mathrm{{g}}}}}{}}) = J^{{{{\mathrm{{g}}}}}{}}, \end{aligned}$$

now takes the physiological interpretation of the longitudinal growth of the individual cardiac muscle cells through the serial deposition of new sarcomere units.

For simplicity, for both transverse and longitudinal growth, we assume stretch-driven growth kinetics,^31^

16 $$\begin{aligned} \dot{\vartheta } = \frac{1}{\tau } \langle \, \lambda - \lambda ^{{{{\mathrm{{crit}}}}}{}} \, \rangle \,, \end{aligned}$$

where the term in the Macaulay brackets ensures that growth is activated only if the current fiber stretch, $\lambda =[\, {\bf{f}}{}_{{{{\mathrm{{0}}}}}{}} \cdot {\bf{F}}{}^{{{{\mathrm{{t}}}}}{}} \cdot {\bf{F}}{} \cdot {\bf{f}}{}_0 \,]^{1/2}$, exceeds the physiological stretch limit $\lambda ^{{{{\mathrm{{crit}}}}}{}}$. We calculate $\lambda ^{{{{\mathrm{{crit}}}}}{}}$ as a regionally varying baseline stretch under physiological conditions. The parameter $\tau$ is a scaling parameter in time, which we are currently calibrating through a series of experiments in chronic porcine models of concentric and eccentric hypertrophy. To individualize the model, we will eventually replace the simple linear growth kinetics (16) by a more sophisticated kinetic equation, e.g., to limit maximum growth,^32^,^53^ or tie the growth process more closely to underlying subcellular mechanisms.^9^ For now, we model growth within a normalized time interval, which corresponds to a physical time interval of months to years.

### Computational Model

We implement the finite growth model as a user defined subroutine into the non-linear finite element program Abaqus/Standard version 6.13.^1^ We represent growth through an internal variable, either $\vartheta ^{\perp }$ or $\vartheta ^{||}$, and store the current growth state locally on the integration point level. To evolve the growth multiplier in time, we adopt a finite difference approximation of the first order time derivative, $\dot{\vartheta } = [\vartheta - \vartheta _{{{{\mathrm{{n}}}}}{}} ] / \Delta t$, where $\vartheta _{{{{\mathrm{{n}}}}}{}}$ denotes the growth multiplier of the previous time step and $\Delta t = t - t_{{{{\mathrm{{n}}}}}{}}$ is the current time increment. The simplified format of the evolution law (16) allows us to explicitly update the growth multiplier of the current time step as $\vartheta = \vartheta _{{{{\mathrm{{n}}}}}{}} + \langle \, \lambda - \lambda ^{{{{\mathrm{{crit}}}}}{}} \, \rangle \, \Delta t /\tau$. We can then calculate the elastic tensor ${\bf{F}}{}^{{{{\mathrm{{e}}}}}{}}$ using either Eqs. (11) or (14), calculate the elastic Jacobian $J^{{{{\mathrm{{e}}}}}{}}$ and the isochoric elastic Green-Lagrange strain tensor $\overline{{\bf{E}}{}}{}^{{{{\mathrm{{e}}}}}{}}$ using Eq. (4), and evaluate the stresses using Eq. (8). Rather than working with the second Piola Kirchhoff stress ${\bf{S}}{}$, Abaqus/Standard uses the Cauchy stress ${\bf{\sigma }}{} = {\bf{F}}{} \cdot {\bf{S}}{} \cdot {\bf{F}}{}^{{{{\mathrm{{t}}}}}{}} / J$. In addition, it requires the Jaumann rate of the Kirchhoff stress ${\bf{\tau }}{}=J \ {\bf{\sigma }}{}$ to ensure optimal quadratic convergence of the Newton-Raphson procedure during the global equilibrium iteration.^56^

### Constitutive Model

Table 1 summarizes the material parameters of our anisotropic constitutive model for myocardial tissue.^22^

**Table 1:** Material parameter values of the healthy human heart determined through inverse finite element analysis using *in vivo* data.^14^

Elastic constants	*D*_0_	kPa	0.001
	*C*_0_	kPa	0.115
Normal stiffness weights	*B*_ff_	–	14.4
	*B*_ss_	–	5.76
	*B*_nn_	–	5.76
Shear stiffness weights	*B*_fs_	–	5.04
	*B*_fn_	–	5.04
	*B*_sn_	–	2.88

Since the commonly used *ex vivo* parameter values for cardiac tissue typically fail to accurately reproduce the* in vivo* response,^2^ we adopt the averaged patient-specific material parameter values from an inverse finite element analysis of five healthy human hearts, three male and two female, age 36 ± 11 years.^14^ Specifically, we use a transversely isotropic version of the Fung model,^22^ and further reduce the model to two independent parameters, the stiffness parameter *C*_0_ and the nonlinearity parameter *B*_0_. We scale the fiber stiffness to $B_{{{{\mathrm{{ff}}}}}{}}=B_0$, the transverse stiffnesses to $B_{{{{\mathrm{{ss}}}}}{}}=B_{{{{\mathrm{{nn}}}}}{}}=0.4 \,B_0$, and the shear stiffnesses to $B_{{{{\mathrm{{sn}}}}}{}}=0.2\,B_0$ and $B_{{{{\mathrm{{fs}}}}}{}}=B_{{{{\mathrm{{fn}}}}}{}}=0.35 \, B_0$.^14^ Using five subject-specific sets of magnetic resonance images, we identify the two parameters *C*_0_ and *B*_0_ through an optimization algorithm with two nested loops: in the outer loop, we optimize *B*_0_ by minimizing the distance of the simulated subject-specific passive pressure–volume response to the Klotz curve, from beginning to end diastole^34^; in the inner loop, we optimize *C*_0_ for a fixed *B*_0_ by minimizing the distance between the simulated end-diastolic pressure and an assumed end-diastolic pressure of 9 mmHg.^14^ The parameter identification results in $C_0=0.115\pm 0.008$ kPa and $B_0=14.4\pm 3.2$, which suggests that the parameter variability in healthy subjects is rather moderate, see Table 1.

### Living Heart Model

Figure 1 illustrates the Living Heart model, an anatomically accurate four-chamber model of the healthy human heart, that provides the basis for our simulation.^5^ Figure 1, right, shows the underlying anatomic model created from magnetic resonance images of a healthy, 21-year old, 50th percentile U.S. male (Zygote Media Group, Inc.; American Fork, Utah). Images were reconstructed from 0.75 mm thick slices using a medium soft-tissue kernel with retrospective electrocardiogram gating.^61^ Data acquisition and reconstruction were performed during 70% diastole. Specifically, images were reformatted to obtain the axial, short axis, vertical long, and horizontal axis. The resulting DICOM data were exported as JPEG files for image post-processing. To create a high-resolution polygonal mesh, the heart tissue was segmented using Amira (FEI; Hillsboro, Oregon). To remove anomalies and scan artifacts, the resulting multi-million polygon mesh was further post-processed using Maya (Autodesk, Inc.; San Rafael, California). From the refined polygonal mesh, NURBS surfaces were created and converted into a solid model using SolidWorks (Dassault Systèmes; Waltham, Massachusetts). The interpolation error between the NURBS surface model and the original segmented polygons was confirmed to be less than 1 mm.^61^ Figure 1, middle left, illustrates the resulting geometric model of the heart with all four chambers, the left and right atria and the left and right ventricles, connected by the four valves.^60^ The tricuspid and mitral valves connect the right and left atria to the right and left ventricles; the pulmonary and aortic valves connect the right and left ventricles to the pulmonary and systemic circulation.

Figure 1, middle right, illustrates the finite element model of the heart discretized with 208,561 linear tetrahedral elements and 47,323 nodes. To create the finite element model, we import the SolidWorks NURBS surface model as solid geometry into Abaqus/CAE (SIMULIA, Dassault Systèmes; Providence, Rhode Island) and generate the tetrahedral discretization. This discretization introduces 141,969 degrees of freedom for the vector-valued deformation ${\bf{\varphi }}{}$ and 208,561 internal variables for the scalar-valued growth multiplier $\vartheta$.^5^

Figure 1, right, shows the muscle fiber model with 208,561 discrete fiber and sheet directions ${\bf{f}}{}_0$ and ${\bf{s}}{}_0$. The muscle fiber vectors wrap helically around the heart, the sheet vectors points transmurally outward.^5^,^59^ To fix the heart in space, we apply homogeneous Dirichlet boundary conditions at the geometric centers of the in- or outlets of all blood vessels. To prescribe different pressure values in each chamber, we model all valves in a fully closed state. Subject-specific geometries extracted from medical imaging are never entirely unloaded; they are subjected to residual stresses. To preload the geometry with a physiological residual stress field, we could adopt the continuum theory of fictitious configurations combined with a fixed-point iteration.^15^ Here, for simplicity, we assume that the geometry is in the end-diastolic configuration and stress free.

### Hypertrophy Model

We model the time line of four pathologies through a combination of concentric and eccentric hypertrophy triggered by left and right ventricular overload. To identify the growth threshold $\lambda ^{{{{\mathrm{{crit}}}}}{}}$ in Eq. (16), we first train the model with its baseline conditions: To simulate the physiological end-diastolic state, we apply a left ventricular and atrial pressure of 5 mmHg and a right ventricular and atrial pressure of 2 mmHg. For each integration point, we record the physiological fiber stretch $\lambda =[\, {\bf{f}}{}_{{{{\mathrm{{0}}}}}{}} \cdot {\bf{F}}{}^{{{{\mathrm{{t}}}}}{}} \cdot {\bf{F}}{} \cdot {\bf{f}}{}_0 \,]^{1/2}$ and store it locally as the growth threshold $\lambda ^{{{{\mathrm{{crit}}}}}{}}$. To model systemic overload, we double the left ventricular and atrial pressure towards an end-diastolic pressure of 10 mmHg, while keeping the right ventricular and arterial pressure at their baseline value of 2 mmHg. To model pulmonary overload, we double right ventricular and atrial pressure towards an end-diastolic pressure of 4 mmHg, while keeping the left ventricular and arterial pressure at their baseline value of 5 mmHg. For the two overload scenarios, we gradually increase the pressure, keep it at its maximum value to allow the ventricles to grow, and then unload the heart. We only consider growth in the ventricles, not in the atria and main blood vessels. For both overload scenarios, we explore the effects of concentric and eccentric hypertrophy.

**Figure 2:**
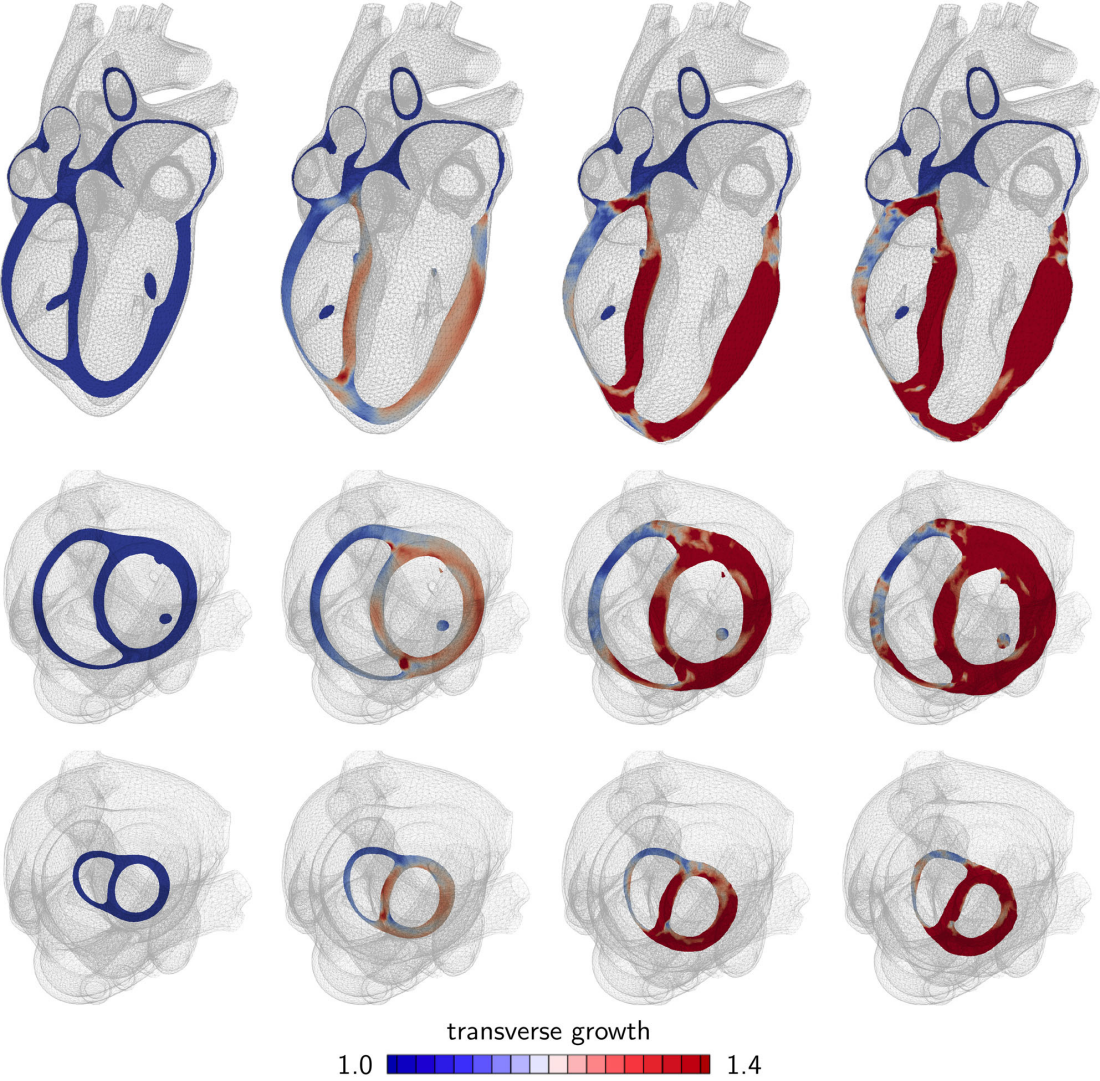
Development of concentric hypertrophy in response to left ventricular overload as predicted by the parallel sarcomere deposition model. Long axis view (top), basal slice of short-axis views (middle) and apical slice of short-axis view (bottom). The color code visualizes the relative thickening of heart muscle cells through parallel sarcomere deposition. Ellipticity is preserved, but wall thickness is drastically increased, which is characteristic for systemic hypertension.

**Figure 3:**
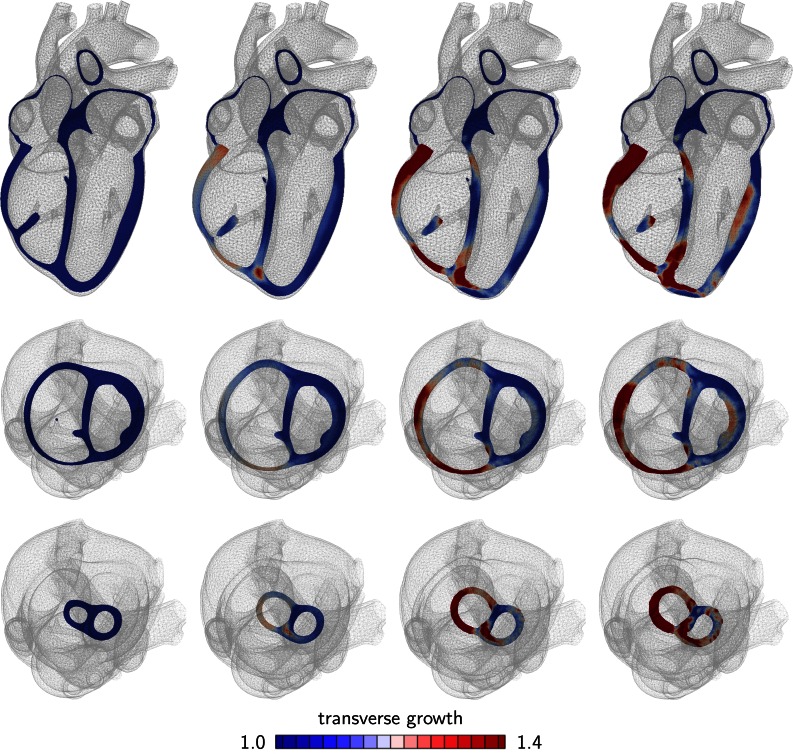
Development of concentric hypertrophy in response to right ventricular overload as predicted by the parallel sarcomere deposition model. Long axis view (top), basal slice of short-axis views (middle) and apical slice of short-axis view (bottom). The color code visualizes the relative thickening of heart muscle cells through parallel sarcomere deposition. Ellipticity is preserved, but wall thickness increases and septum curvature reverses, which is characteristic for pulmonary hypertension.

## Results

### Concentric Hypertrophy

Figures 2 and 3 show the development of concentric hypertrophy in response to left and right ventricular overload as predicted by the parallel sarcomere deposition model. The snapshots summarize the evolution of hypertrophy at four time points and three viewpoints: one long-axis view, top row, and two short-axis views, one basal slice, middle row, and one apical slice, bottom row. The color code illustrates the relative thickening of the individual heart muscle cells through myofibrillogenesis, the parallel deposition of sarcomere units. The value of $\vartheta ^{\perp }=1.0$ indicates no thickening; the maximum value of $\vartheta ^{||}=1.4$ indicates a cardiomyocyte thickening of 40% through the addition of 40% more myofibrils in parallel to the cell’s long axis.

Figure 2 illustrates the typical characteristics of systemic hypertension: an increase in left ventricular wall thickness at a relatively constant overall size. The short axis view visualizes the classical secondary effects associated with left ventricular wall thickening: a narrowing of the ventricular cavity and a decrease in left ventricular volume associated with an impaired diastolic filling.

Figure 3 documents the features of pulmonary hypertension: an increase in right ventricular wall thickness and a pronounced inversion of septal curvature.

**Figure 4:**
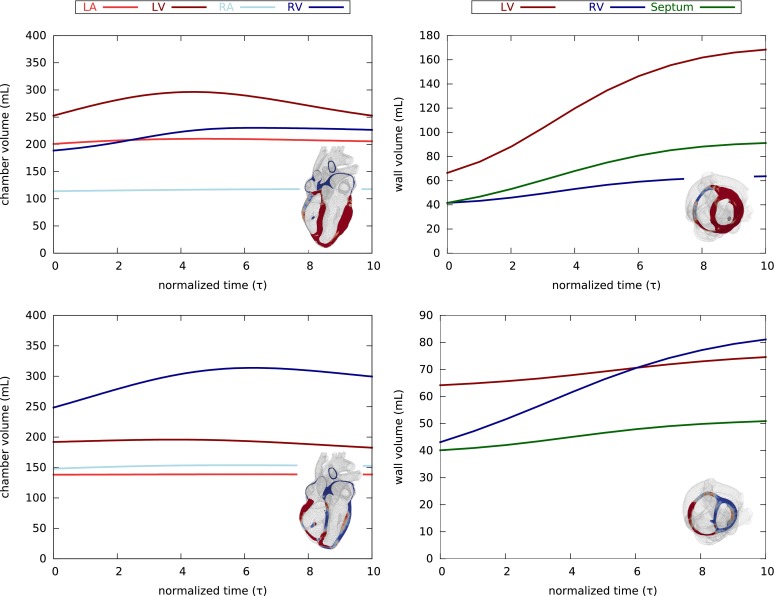
Evolution of cardiac chamber and wall volumes during concentric hypertrophy in response to left (top) and right (bottom) ventricular overload as predicted by the parallel sarcomere deposition model. Chambers volume increase slightly at first, and then decrease as the walls thicken (left). Wall volumes increase drastically, but then saturate as the wall stretches return to their physiological baseline values (right).

Figure 4 summarizes the evolution of the cardiac chamber and wall volumes during concentric hypertrophy in response to left and right ventricular overload. The chamber volume of the overloaded chamber increases at first, and then decrease as the wall thickens. The wall volumes increase rapidly at first, but then saturate as the wall stretches return to their physiological baseline values.

### Eccentric Hypertrophy

**Figure 5:**
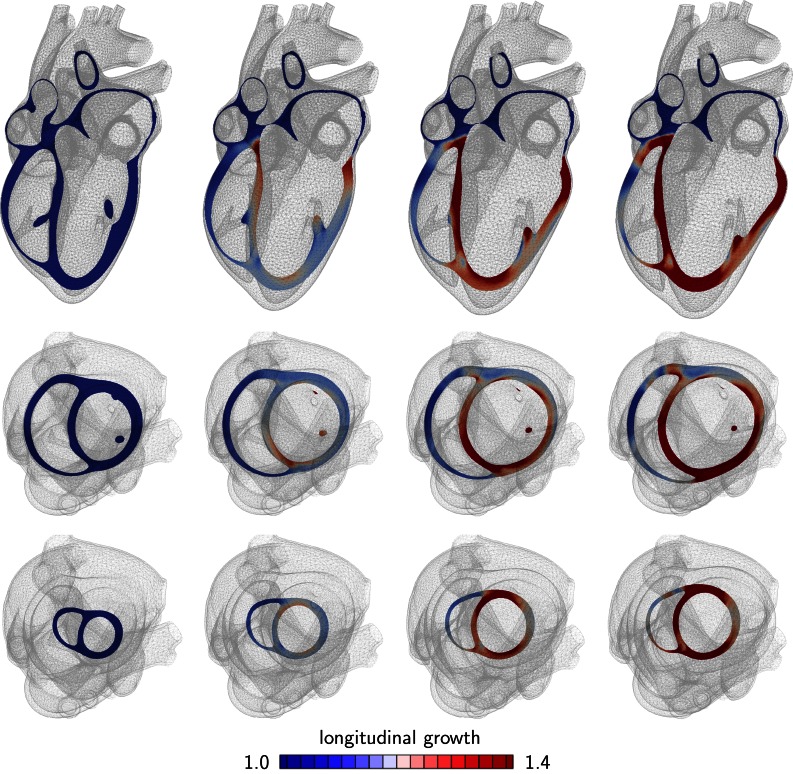
Development of eccentric hypertrophy in response to left ventricular overload as predicted by the serial sarcomere deposition model. Long axis view (top), basal slice of short-axis views (middle) and apical slice of short-axis view (bottom). The color code visualizes the relative lengthening of heart muscle cells through serial sarcomere deposition. The increase in left ventricular volume and loss of ellipticity are classical features of left heart failure.

**Figure 6:**
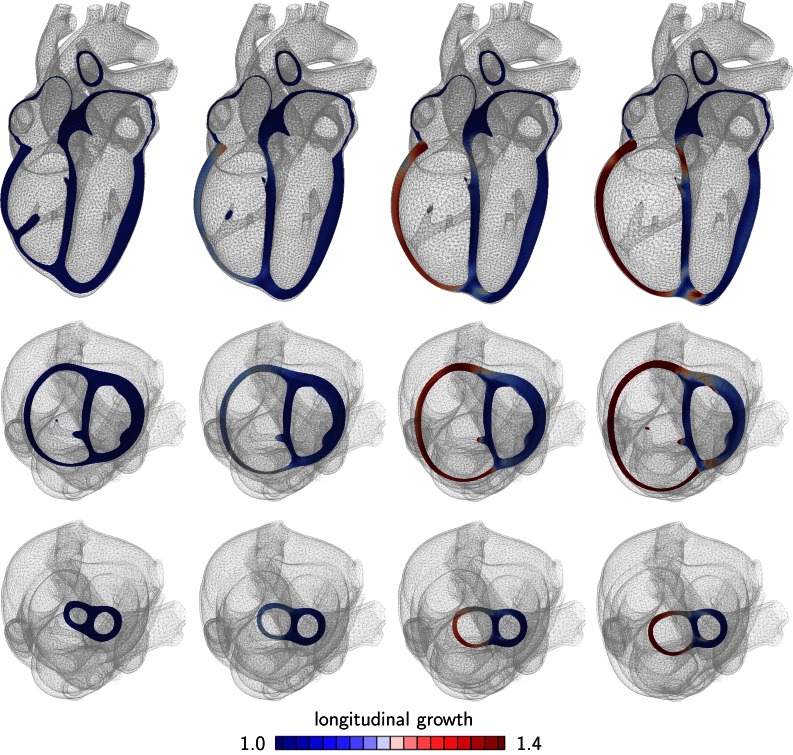
Development of eccentric hypertrophy in response to right ventricular overload as predicted by the serial sarcomere deposition model. Long axis view (top), basal slice of short-axis views (middle) and apical slice of short-axis view (bottom). The color code visualizes the relative lengthening of heart muscle cells through serial sarcomere deposition. Reverse curvature of the septum and D-shape cross section of the left ventricle are classical features of right heart failure.

Figures 5 and 6 show the development of eccentric hypertrophy in response to left and right ventricular overload as predicted by the serial sarcomere deposition model. The snapshots summarize the gradual progression of hypertrophy at four time points and three viewpoints: one long-axis view, top row, and two short-axis views, one basal slice, middle row, and one apical slice, bottom row. The color code illustrates the relative lengthening of the individual heart muscle cells through sarcomerogenesis, the serial deposition of sarcomere units. The value of $\vartheta ^{||}=1.0$ indicates no lengthening; the maximum value of $\vartheta ^{||}=1.4$ indicates a cardiomyocyte lengthening of 40% through the addition of 40% more sarcomeres along the cell’s long axis.

Figures 5 illustrates the characteristic features of left heart failure: a progressive increase in left ventricular volume at a relatively constant wall thickness and a gradual transition from elliptical to spherical shape. Hypertrophy starts first at the endocardium, the inner wall, and then propagates toward the epicardium, the outer wall. The long-axis view reveals the classical secondary effects associated with ventricular dilation: papillary muscle dislocation and mitral annular dilation.

Figure 6 illustrates the characteristic features of right heart failure: an increase in right ventricular volume at a relatively constant wall thickness and a decrease in septal curvature associated with a change in left ventricular cross section from circular to D-shaped size.

**Figure 7:**
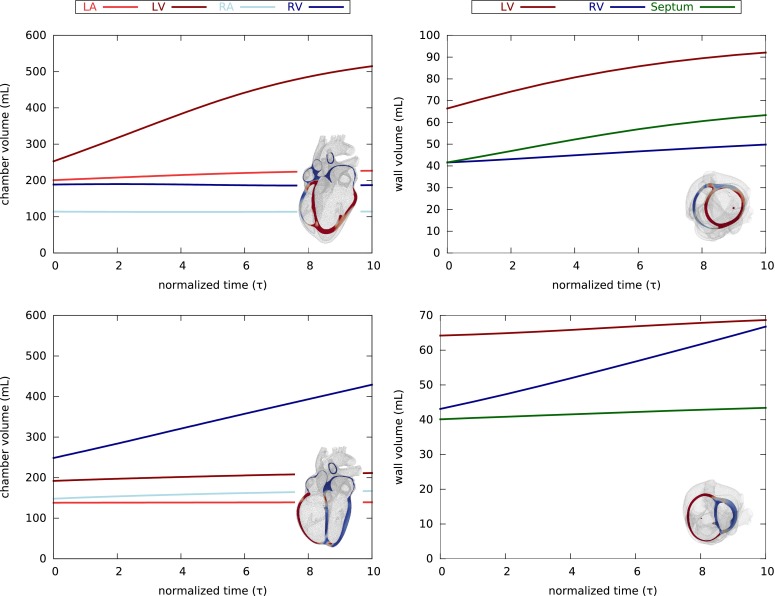
Evolution of cardiac chamber and wall volumes during eccentric hypertrophy in response to left (top) and right (bottom) ventricular overload as predicted by the serial sarcomere deposition model. Chamber volumes increase drastically with only minor saturation (left). Wall volumes increase moderately and do not saturate as the ventricles continue to dilate (right).

Figure 7 summarizes the evolution of cardiac chamber and wall volumes during eccentric hypertrophy in response to left and right ventricular overload. For both cases of overload, the volume of the overloaded chamber increases drastically, while all other chamber volumes are only marginally affected. The wall volume of the affected chamber increases gradually as the ventricle continues to dilate. In both cases, left and right ventricular overload, eccentric hypertrophy continues for continuing overload. Compared to the parallel sarcomere deposition model in Fig. 4, wall volumes increase less drastically for the serial sarcomere deposition model in Fig. 7.

**Figure 8:**
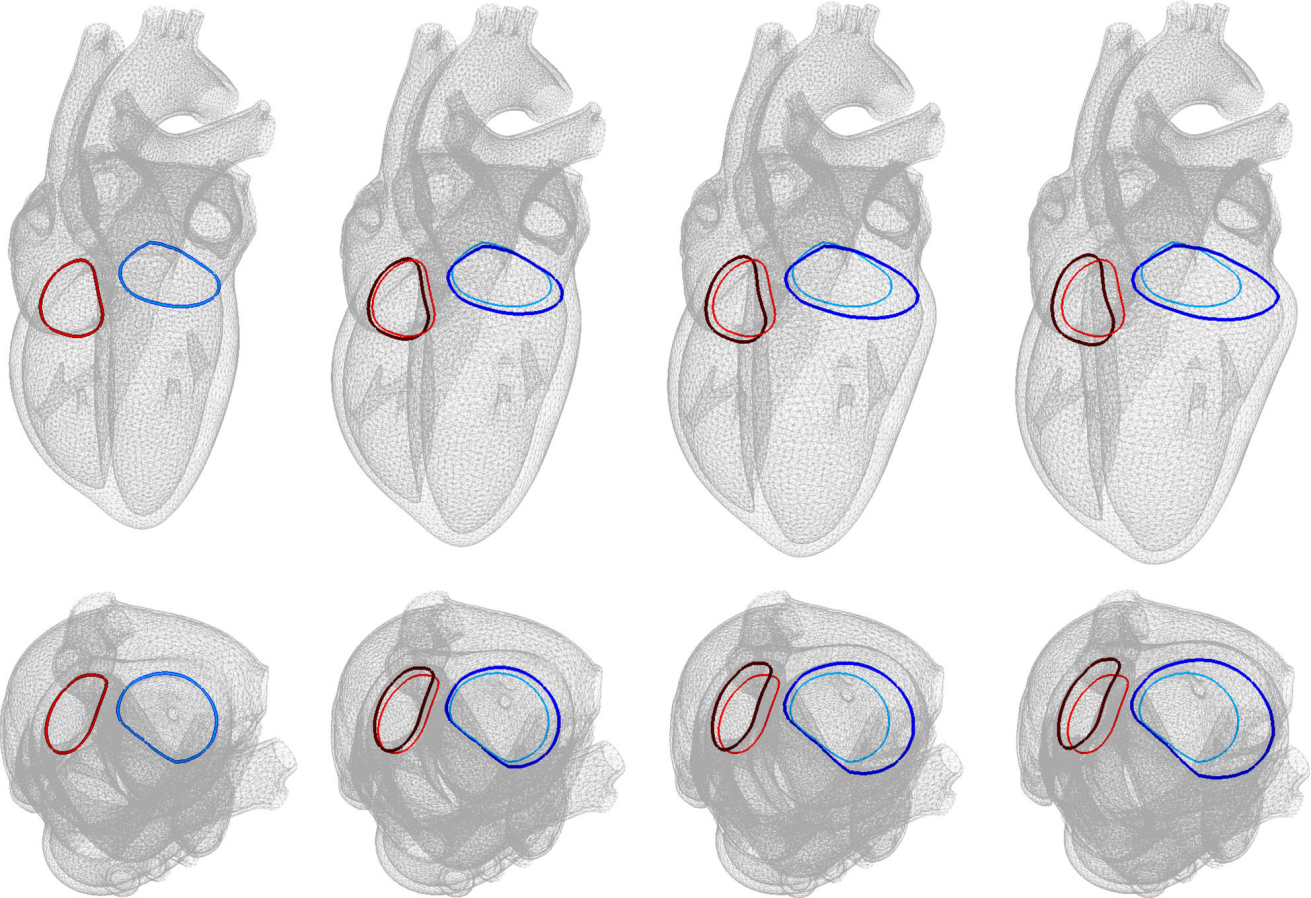
Development of annular dilation in response to left ventricular overload as predicted by the serial sarcomere deposition model. Long axis view (top) and short-axis views (bottom). The colors visualize the tricuspid and mitral annuli. Mitral annular dilation can lead to mitral regurgitation and is a classical secondary effect of left ventricular overload.

**Figure 9:**
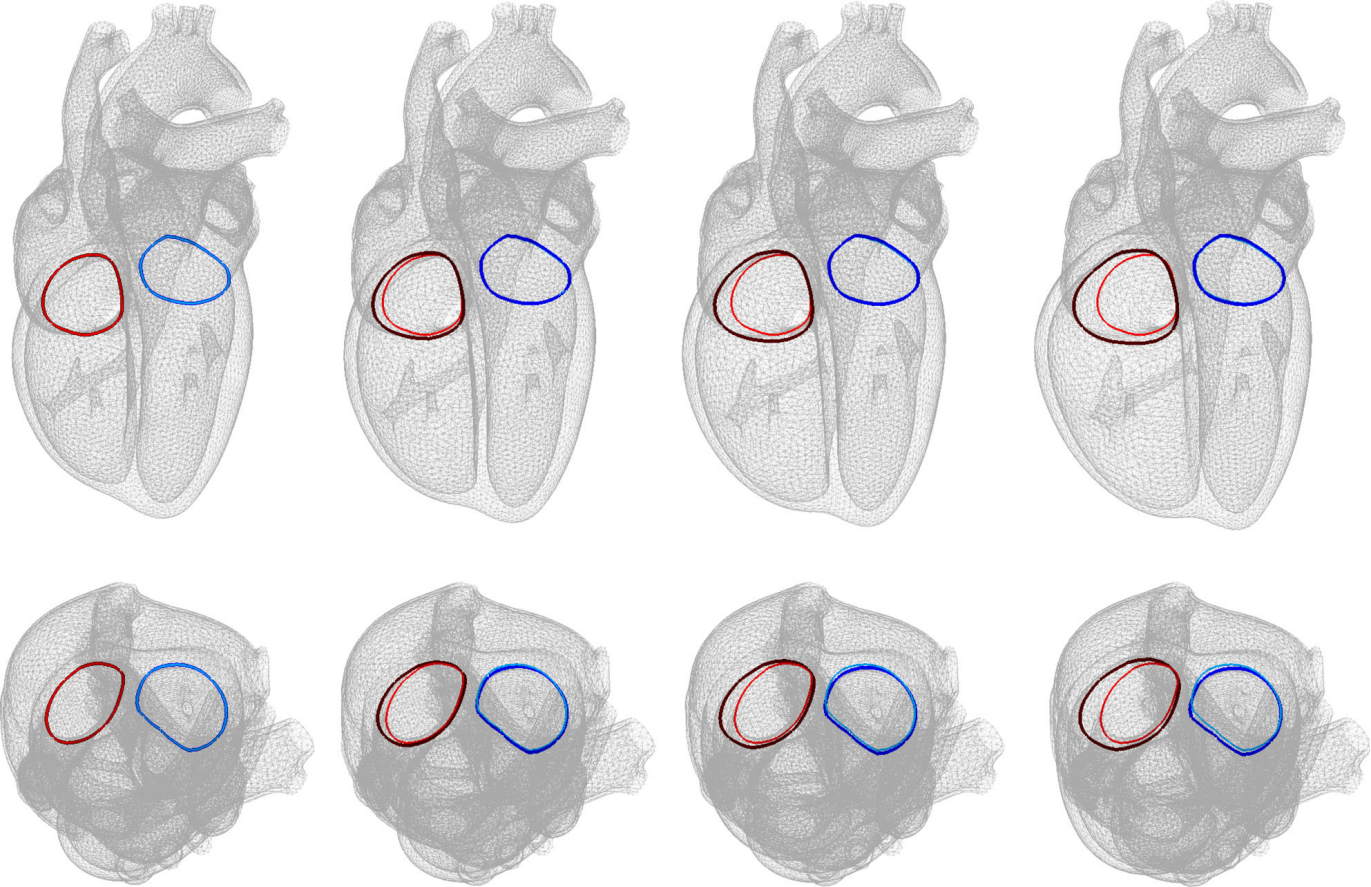
Development of eccentric hypertrophy in response to right ventricular overload as predicted by the serial sarcomere deposition model. Long axis view (top) and short-axis views (bottom). The colors visualize the tricuspid and mitral annuli. Tricuspid annular dilation can lead to tricuspid regurgitation and is a classical secondary effect of right ventricular overload.

Figures 8 and 9 show the development of annular dilation in response to left and right ventricular overload. The colors visualize the tricuspid and mitral annuli. Left ventricular overload causes a pronounced mitral annular dilation, which can eventually lead to mitral regurgitation, a classical secondary effect of left ventricular overload. Right ventricular overload causes tricuspid annular dilation, which can lead to tricuspid regurgitation, a classical secondary effect of right ventricular overload.

**Figure 10:**
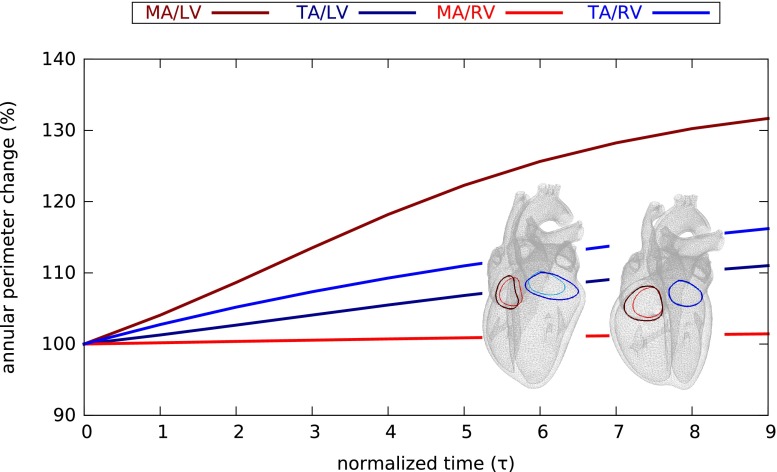
Evolution of annular perimeter during eccentric hypertrophy in response to left and right ventricular overload as predicted by the serial sarcomere deposition model. The mitral annular perimeter increases drastically upon left ventricular overload (dark red) but remains unaffected by right ventricular overload (light red); the tricuspid annular diameter increases moderately upon left ventricular overload (dark blue), but increases markedly upon right ventricular overload (light blue).

Figure 10 quantifies the evolution of the mitral and tricuspid annular perimeters during eccentric hypertrophy in response to left and right ventricular overload. The mitral annular perimeter increases drastically upon left ventricular overload, but remains unaffected by right ventricular overload. The tricuspid annular diameter increases moderately upon left ventricular overload, but increases markedly upon right ventricular overload.

**Figure 11:**
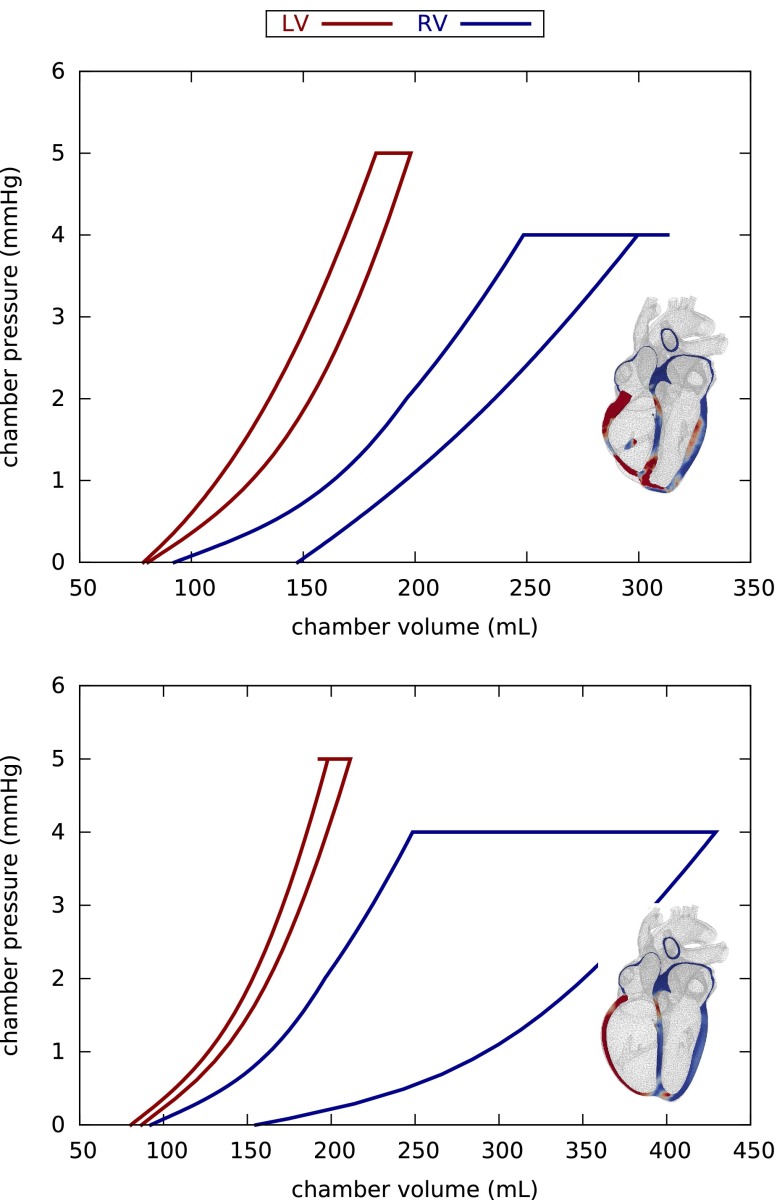
Pressure–volume relationships during concentric (top) and eccentric (bottom) hypertrophy in response to right ventricular overload as predicted by the parallel and serial sarcomere deposition models. Both growth laws induce residual stress in the overloaded right ventricle. Concentric growth decreases ventricular compliance; eccentric growth increases ventricular compliance.

Figure 11 illustrates an interesting difference between concentric and eccentric hypertrophy, the evolution of the pressure–volume relationships during right ventricular overload. During concentric growth, the right ventricular pressure–volume loop shifts to the left indicating an increase in right ventricular stiffness. During eccentric growth, the right ventricular pressure–volume loop shifts to the right indicating an increase in right ventricular compliance. This is in general agreement with the common understanding that the heart stiffens during hypertrophic cardiomyopathy—a classical hallmark of hypertension—and softens during dilated cardiomyopathy—a classical hallmark of heart failure.

## Discussion

Patient-specific modeling holds promise to optimize treatment on an individual, personalized basis. Existing patient-specific models can now reproduce the physiological behavior of the living heart and predict the acute, immediate response to changes in the mechanical environment. These changes can be induced naturally, for example through pressure or volume overload, or interventionally, through surgery or other types of treatment. However, unfortunately, most existing models fail to predict the chronic, long-term response to environmental changes. This implies that we can not yet make reliable statements about the timeline of a progressive disease or about the long-term success of a treatment option. Here we establish a new generation of whole heart models to predict the acute and chronic pathophysiology of the living heart for the examples of concentric and eccentric hypertrophy.

While previous phenomenological growth models prescribe an assumed rule to drive the remodeling process.^37^ here growth is a natural consequence of overload: in concentrical hypertrophy, growth is self-regulated and converges to a homeostatic equilibrium state; in eccentric hypertrophy, growth continues unboundedly. In contrast to previous models, which prescribe homogeneous growth through a single gradually increasing growth multiplier,^31^ here the growth multiplier is heterogeneous by definition, it evolves naturally, varies with chamber geometry, and depends inherently on the patient-specific overload level. In contrast to previous models, in which the homeostatic state is prescribed by a single phenomenological baseline stretch $\lambda ^{{{{\mathrm{{crit}}}}}{}}$^17^ or baseline stress $\sigma _0$,^8^ here we calculate the homeostatic state as the regionally varying response under patient-specific baseline conditions. This implies that the baseline stretch $\lambda ^{{{{\mathrm{{crit}}}}}{}}$ is no longer an arbitrary parameter—it follows naturally from individual physiological conditions.

Concentric hypertrophy is characterized through an increase in wall thickness at a relatively constant cardiac size.^39^ Our simulations in Figs. 2 and 3 naturally capture these pathophysiological features. Typical secondary effects associated with systemic hypertension are a decrease in chamber volume and a potential outflow obstruction through pronounced septal growth.^44^ Under chronic conditions, these geometric changes might eventually impair diastolic filling, reduce cardiac output,^28^ and decrease the overall blood supply to the body.^10^ While single- or bi-ventricular models are able to reproduce the decrease in chamber volume,^37^,^51^ they might be insufficient to make predictions about the impact of these changes on outflow characteristics.^44^. The long axis views in Fig. 2 demonstrate that our four-chamber heart model naturally captures chronic alterations in septal wall geometry and their impact on the outflow tract of the left ventricle. Typical secondary effects associated with pulmonary hypertension are a reduction of septal curvature^50^ and a change in left ventricular cross section from circular to D-shaped size.^23^,^24^ Under chronic conditions, these geometric changes may induce abnormal septal function, and impair left ventricular performance through ventricular interdependence. The short axis views in Fig. 3 illustrate that our model is capable of predicting these characteristic effects.

Concentric hypertrophy is characterized through a negative feedback loop: wall thickening is a compensatory mechanism to accommodate an increase in pressure; it is a self-regulatory process that converges towards a new homeostatic equilibrium state at a higher pressure level. According to the law of Laplace, the wall stress decreases as the wall thickens. This implies that the stretch, our driving force for growth, gradually returns to its physiological baseline value. This deactivates further growth as predicted by our model in Fig. 4. The compensatory thickening of the ventricles at a relatively constant ventricular size—a classical hallmark of systemic and pulmonary hypertension—is in agreement with our model predictions.

Eccentric cardiac hypertrophy is characterized through a progressive increase in cardiac diameter at a relatively constant wall thickness,^39^ and a gradual transition from elliptical to spherical shape.^11^ Our simulations in Figs. 5 and 6 naturally capture these pathophysiological features. Typical secondary effects associated with left ventricular dilation are a dislocation of the papillary muscles^20^ and an increase in mitral annular area.^51^ Under chronic conditions, these geometric changes might lead to mitral regurgitation,^12^ tricuspid regurgitation,^54^ reduced ejection fraction,^10^ and insufficient blood supply to the body.^52^ While changes in mitral and tricuspid annular geometry are often insufficiently captured in single- and bi-ventricular models,^18^ Figs. 8 and 9 demonstrate that our four-chamber model is capable of predicting these effects. Our simulations of annular dilation could have important implications in selecting and sizing annuloplasty bands or rings to reduce mitral or tricuspid regurgitation.

Eccentric cardiac hypertrophy is characterized through a positive feedback loop: ventricular dilation increases progressively with continuing overload. According to the law of Laplace, the ventricular wall stress is proportional to the ventricular pressure and the ventricular radius, and inversely proportional to the ventricular wall thickness. This implies that the end-diastolic fiber stress, the fiber stretch, and the overall chamber volume keep increasing as predicted in Fig. 7. Overall, the progressive dilation of the ventricles at a relatively constant wall thickness—a classical hallmark of heart failure—is in excellent agreement with our model predictions.

To address the inherent limitations of our model, the next natural steps are two-fold: On the cellular level, the next step will be to establish, calibrate, and validate more mechanistic growth laws,^32^ which accurately represent the individual signaling pathways associated with concentric and eccentric growth.^25^ On the whole organ level, the next step will be to streamline the integration of patient-specific data,^36^ including individual hemodynamics and chamber geometries.^2^ Parameterized in terms of hierarchically organized NURBS surfaces,^61^ our healthy four-chamber heart model has the potential to serve as a template, which could be individualized* via* morphing using specific anatomic landmarks of individual patients. Coordinated efforts along these lines will shape the way towards personalized diagnostics and decision making.^30^

In summary, our simulations of concentric and eccentric hypertrophy agree favorably with the clinical observations in patients with left and right ventricular overload. Our model naturally connects the molecular events of parallel and serial sarcomere deposition with cellular phenomena of myofibrillogenesis and sarcomerogenesis to model organ level function during concentric and eccentric hypertrophy. Unlike most existing models for cardiac maladaptation, our model not only accounts for the end points of the disease, but enables the prediction of disease progression with its characteristic negative and positive feedback loops. The Living Heart model, an anatomic model of the human heart with all four chambers and valves, not only allows us to predict chronic alterations in wall thickness, chamber size, and cardiac geometry, but also to quantify the long-term effects of these alterations on papillary muscle position, valvular geometry, regurgitant flow, and outflow obstruction. To calibrate our model, we are currently performing a series of experiments to characterize the regional and temporal progression of concentric and eccentric hypertrophy in chronic porcine models of pressure and volume overload. Upon appropriate calibration, our model has the potential to link cell level characteristics, e.g., an increase in the serial sarcomere number, to whole organ form and function, e.g., an increase in end-diastolic volume and a decrease in ejection fraction, with the ultimate goal to estimate the risk of heart failure and support decision-making on an individualized, patient-specific basis.
